# Smart Metering Systems Optimization for Non-Technical Losses Reduction and Consumption Recording Operation Improvement in Electricity Sector

**DOI:** 10.3390/s20102947

**Published:** 2020-05-22

**Authors:** Ilie Vlasa, Adrian Gligor, Cristian-Dragos Dumitru, Laszlo Barna Iantovics

**Affiliations:** 1 Electricity Distribution Company Mures Branch, S.D.E.E. Transilvania Sud S.A., Târgu Mureș 540320, Romania; ilie.vlasa@distributie-energie.ro; 2 Department of Electrical Engineering and Information Technology, “George Emil Palade” University of Medicine, Pharmacy, Science and Technology of Targu Mures, Târgu Mureș 540088, Romania; cristian.dumitru@umfst.ro

**Keywords:** electricity sensor, smart grid, metering device, optimization, electricity consumption measuring, misconduct electricity usage

## Abstract

One of the keys of enhancing the quality of electric power supply resides in the accuracy of the consumption metering. Nowadays development of the sensors, devices and systems for electricity metering offers the basis for this service. Nevertheless, this achievement in many situations is altered such that appropriate measures must be adopted even if already significant costs have been registered. In this paper is proposed and discussed an optimal solution based on the identification and minimizing the measurement errors for increasing the electricity readings accuracy and lowering the electricity losses and related costs. In this regard, a mathematical model was developed and a particular algorithm for the mentioned problem is proposed and tested in the case of a power distribution company where an enhancement on average of the own technological consumption with 4% was recorded.

## 1. Introduction

Electricity is one of the most important products of contemporary society, practically making possible the development of many and complex industrial or residential human activities. In order to ensure the efficiency of the production, transport, distribution and use of electricity, it became necessary to monitor and record its parameters as accurate and actual (in real time) as possible [1,2,3]. The interest of stakeholders (producers, transporters, distributors, suppliers and consumers) was also supported by the development of legislation and regulations both nationally and internationally. These refer to programs and directives for the implementation of advanced digital measurements and recording systems of electricity. For example, at the EU level, directives such as 2009/72/EC [4] or 2019/944 [5] are well known and regard the development of the internal energy market, with an emphasis on ensuring high standards of providing the services in this field, important being in this case an optimistic coverage of at least 80% of consumers with intelligent metering systems (smart meters (SM)), quantified to almost 200 million such devices as [6] states. Regulations in the same direction are also met in the US, for example the Energy Independence and Security Act of 2007 [7], which refers to the modernization of the power system in general and in particular to the adoption of intelligent sensors for measurement. The interest in adopting these solutions shows that if, at the end of 2015, 50% of the domestic consumers were covered (the equivalent of 65 million smart meters), at the end of 2020 the expectation is to reach a threshold of 90 million devices of this type [8], representing an adoption rate of 67% [9]. Furthermore, in some countries of the Middle East a policy of developing intelligent metering systems for electricity is implemented. For example, the Islamic Republic of Iran intends that by 2025 approximately 70% of the metering facilities will be upgraded for remote information transmission [10]. In Asia, in the case of India, the year 2017 represented a milestone for net metering implementation for consumers equipped with roof-top solar system and by 2022 it is intended that consumers from major cities and from some selected urban areas will have the opportunity to chose their electricity suppliers [11]. Countries such as China and Japan also provide substantial incentives for the use of smart meters, while on the South American continent, part of the Middle East and Africa penetration of smart meters is lower [12]. As shown in literature, a global appreciation estimates that by 2020 the number of devices integrated into advanced reading systems providing Advanced Metering Infrastructure (AMI) will reach the level of 800 million units [12].

The adoption of the new systems has been and will continue to be possible due to the development of technologies in the field of information and communications technology, such as Internet of Things (IoT), Internet of Everything (IoE) technologies, etc.

The important feature that intelligent energy measurement solutions offer is the high measurement accuracy (+/−0.2% compared to +/−2% for many of old sensors) due to the high precision current and voltage sensors, by the digital-analog with high sampling frequencies conversion systems and by digital signal processing and processing features for an improved number of functions such as: calculations of all types of energies on the both delivery and receiver channels, load consumer profile or phasor diagrams.

The basic functions of device interconnection also allow the implementation of new features such as the creation of sensor networks, the connection of sensors with databases in the cloud and the development of distributed control systems that can even be based on intelligent algorithms. In this paper we propose the development of a solution that allows to improve the accuracy of the readings of the energy consumption and implicitly the decrease of the technological costs and the increase of the consumers service quality by an invoicing as equitable as possible and early detection of the problems related to the quality of the service or to the quality of the supplied electricity.

## 2. Context Overview of Smart Meters Rollout in Electricity Sector. The Importance of Smart Metering Systems

**Figure 1:**
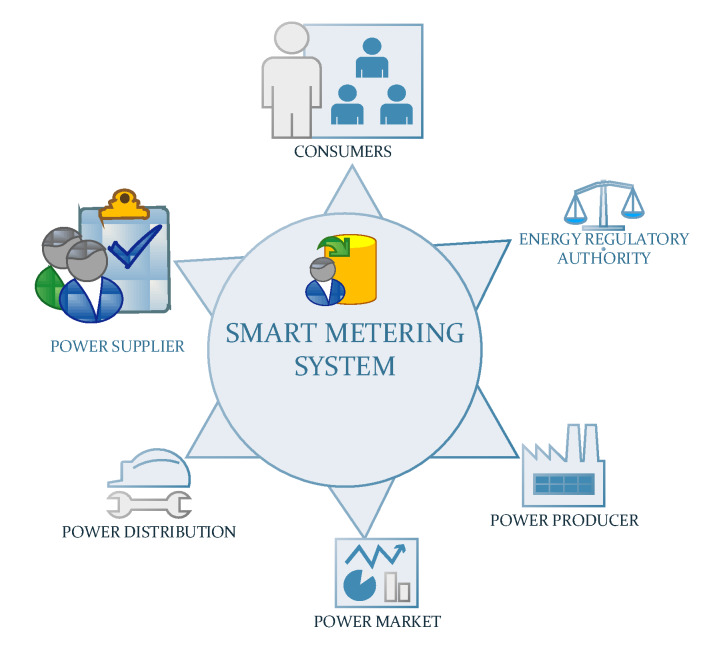
Stakeholders involved in smart metering adoption.

The adoption of smart metering systems is an option that implies both consumers and distributors/suppliers of utilities as stakeholders (Figure 1) which are currently based on the interest of reducing costs and losses with these utilities as well as to use and manage these consumptions more efficiently.

### 2.1. Adoption Factors of Intelligent Sensors for Measurement Related to Cost Reduction

One of the factors that influence consumers’ choices and their actions is the costs of the services or of the products purchased. In the case of electricity, the consumers’ confidence regarding the measurement with the smallest deviations and the possibility of tracking the consumption are the most relevant factors that can determine their decisions. The generalized computerization correlated with the accessibility of the necessary technologies increased the interest of the users for the tracking of the registered electricity consumption. The solution to this requirement came through the introduction in the electricity sector of smart metering systems. These, through the technologies offered and the mode of operation (a better measurement accuracy, values reading at shorter intervals, distance reading, that lead to the decrease of the consumer inconveniences) has attracted the consumers’ attention and increased the interest for their adoption in the place of the classic measurement methods. The preference of many users for new technologies, as a subjective aspect, cannot be neglected here.

On the other hand, the development of the energy market and the energy policies regarding the optimal operation of the power system have led to the introduction of measures, seen as means of intervention, that will allow the users to co-interest and to adapt their behavior from the consumer position in accordance with the proposed objectives. One of the popular means is to introduce the differentiated hourly tariff for electricity, which is only possible if smart metering solutions are used.

The liberalization of the energy market in many regions of the world allows consumers to optimize energy costs by choosing the suppliers with the most competitive prices or in some situations according to the primary energy source directly or by through tradable commodity such as green certificates [13,14]. Operation in this context is complicated or sometimes impossible in the case of classical measurement systems. The adoption of smart metering systems allows the easy change of the energy supplier by rapidly and efficiently recording the consumption according to the contracts between the involved stakeholders.

### 2.2. Adoption Factors of Intelligent Sensors for Measurement Related to Loss Reduction

The efficient use of electricity becomes an important factor for both developed and developing countries. This also includes how electricity supplies consumers at least from the perspective of economic efficiency. Considering this aspect, electricity distributors focus on the factors that influence electricity losses in the transmission and distribution networks [15], regarded as technical losses or commercial losses. The technical losses occur naturally and are caused by the dissipation of electricity in the power lines and in equipments used for the production, transportation and distribution of electricity. The most significant losses (about 90% of the total losses) are encountered in the power circuits due to the conductors heating by Joule effect at the electric current flow through the electrical circuits [16], but there are also losses caused by the corona effect as well as by the imperfect insulation within the transport and distribution installations [17]. These losses can be highlighted by the energy balances performed that are based on the measurements provided by the SM systems or can be estimated by algorithmic procedures as presented in [18].

An important role in reducing the Own Technological Consumption (OTC), which results from the difference between the energy entered in the Commercial Contour and the energy distributed to the consumers [19] by any electricity distributor, is represented by the diminution of the non-technical losses. The non-technical losses can be attenuated by increasing the security of the distribution installations, in particular by frequent checking of the measurement groups both for domestic, but especially for industrial consumers [20]. Electricity theft represents an illegal practice to obtain electricity for different uses, which results in significant losses for electricity distribution companies. A loss of about USD 25 billion [15] is estimated worldwide, of which USD 6 billion is estimated to be in the United States, representing around 3.5% of the electricity consumed [21], a loss mainly due to unauthorized manipulation of analog meters. Non-Technical Losses (NTLs), which are usually attributed to the theft of electricity before the metering group (through false columns) or by falsifying the energy meter, by measuring or administering errors and unpaid electricity are recorded with predominance in developing countries such as those in South Africa, where losses of up to 50% of the electricity produced are recorded [22]. A significant loss of electricity due to fraudulent consumption is also recorded in India, where if at least 10% of NTL were recovered it would save approximately 83,000 GWh annually [23]. The smallest non-technical losses (below 6%) are registered in countries such as: Finland, Germany, Holland, Japan, etc. [24], where fraudulent electricity consumption is strongly discouraged by prosecuting individuals or organizations involved.

In addition to economic losses, theft of electricity is also a major issue in terms of people safety (people that live in the vicinity of communities where the theft of electricity has a high percentage of occurrences). For example, in an area of eastern Uganda, where an NTL of over 50% is recorded, approximately 50 people are electrocuted every week [25].

An effective method for stopping unauthorized interventions in electricity distribution facilities is to replace the conventional meters with smart meters [26] that are able to identify electricity consumption in a more detailed way, as well as some events related to the quality of electricity or unauthorized interventions in electrical installations. The smart meters have implemented a number of functions that are beneficial for both the network operator (by assuring an accurate and real-time metering of the delivered electricity), as well as for the consumer (in order to optimize the electricity requirement). Among consumers who have implemented smart telecommunication systems, there are people who have certain objections regarding smart meters because of the opportunity to disclose confidential consumer data, which is not ethically viable; some countries such as The Netherlands or the UK have even given up the implementation of Intelligent Measurement Systems (MS) projects due to data confidentiality issues [27]. Furthermore, there are some consumers “claiming” that the smart meters are a source of dangerous radiations for human life [15]. By taking into consideration practical met aspects, cases of smart meters interfering with radio frequencies that disrupt radio-TV broadcasts were reported or cases of communication of radio-mobile stations disruption used by police and gendarmes, which communicate on the same frequency waves as the smart meter radio module [21]. In order to eliminate the radiation emission, metering systems that use wired communication means (current loop) can be used, which are much more secure and reliable in terms of data communications between power meters and the central communication system. Hybrid systems with wireless communication routes (radio waves) [28] are also available and can be used, but these systems are difficult to implement economically, the equipment used in a hybrid communication system having quite high prices. In order to secure the consumer privacy, a telecommuting system in which each smart meter is identified by a unique ID can be used, which can be found only in the database of the remote management system, where the analogy between this ID and the counter series will be made, as well as the location of the device through which the amount of energy will be billed from that location [29]. Another option is the use of solutions based on encryption algorithms, an example being the solution proposed by Xie et al. in [30].

The consumption monitoring function is already implemented in the case of smart metering, but the metering system can be accompanied by certain additional modules, which can signal in a timely manner the possible voltage or current interruptions in the secondary circuits of the semi-direct or indirect measurement schemes from industrial consumers [31]. In the case of domestic consumers and economic agents, the main non-technical losses come from the failure of the energy meters or bypassing them through false columns. In order to detect these problems, a series of methods are used, such as checking the measurement group together with the connection cable for each consumer, or for real-time monitoring, a supplemental current measurement (based on current transformer) can be fitted on each connection that totals the current passed through the respective circuit (when there is a difference in energy between the mounted device and the meter, through the attached GSM module, the distribution operator is notified) [32]. The two-above mentioned solutions for real-time monitoring of electricity consumption for industrial and household consumers are very effective in terms of real-time detection of possible measurement errors, which may come from failure of the measuring group or due to unauthorized interventions in electrical installations. The solutions described are difficult to implement in the current configuration of electrical networks, as the distribution and measurement boxes are not provided with a specially designed space for these types of equipment. The cost of implementing these additional monitoring systems is quite high, so the investment in these systems is not quite justified.

### 2.3. Current Approaches Regarding Control of the OTC through Unauthorized Energy Theft Minimization

The analysis of the electricity consumption for different types of customers is essential for the Electricity Distribution Operators in order to establish the optimal decisions of operative management and the reduction of the OTC targeting several points of intervention such as: identifying the unauthorized thefts of electricity and quality of the delivered electricity improvement. Usually the electricity consumption is not uniform, it varies from one type of consumer to another, depending on: geographical area, season, weather conditions, day period of time. If we refer to the detection of fraudulent electricity consumption, the scientific literature reports the existence of implementations based on a series of algorithms for analyzing the load curve of each individual consumer. Such a solution proposed in [33,34] is based on the energy quantities recorded in periods (samples) of 15 min, which are compared with a standard load curve adapted to the area and to the consumer group to which the energy customer referred to is part. This algorithm for coding and comparing the load curves of residential consumers identifies on the basis of data analysis accurately the places of consumption where electricity is erroneously recorded, but for the implementation of the specified algorithm it is necessary to purchase consumption data with a sampling period for less than an hour; unfortunately, most smart meters are designed and programmed to record the load curve only hourly and not at every quarter of an hour.

An important factor in reducing fraudulent electricity consumption is the detection of anomalies that occur in the process of electricity metering and billing. The anomalies represent considerable differences between the amounts of energy billed in a given time sequence and the threshold set for the type of consumer chosen. The Lempel–Ziv algorithm presented in [35] could be a universal technique for predicting illegal energy theft, based on techniques for detecting anomalies in the system for measuring and billing electricity. This additional measure effectively helps the electricity distribution operators to identify and penalize the theft of electricity, but there can be no clear difference between the measuring points that record a lower amount of energy due to the lack of activity related to the consumption place, due to the failure of the measurement group or due to bypassing the measurement group by false columns.

## 3. Smart Metering Architecture and Implementation

**Figure 2:**
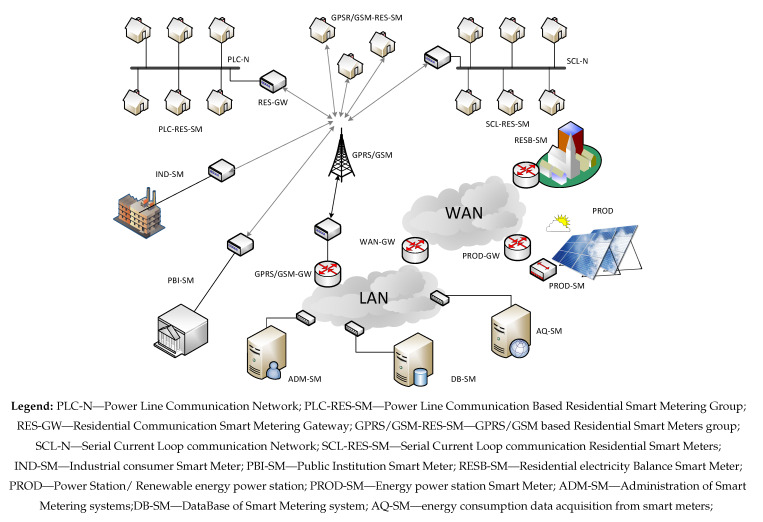
A typical electricity smart metering system structure.

For the implementation of a modern system of electricity distribution it is necessary to replace the classic meters with smart meters that will be mounted in each node of the network: substations, power exchange substations and power distribution substations, as well as at points between branches and transformer stations or at the end points of the power network, for recording energy consumption and reporting it in real time to the network operator [36]. A common modernised structure is synthetized in Figure 2.

### 3.1. Smart Metering Adoption and Specific Operation Issues

Smart electricity meters offer a number of advantages to both the distributor, the supplier and to the electricity consumer, which are summarized in Table 1.

Smart meters significantly simplify the process of measuring and collecting data for each consumer but they are also subject to cyber attacks [37], as most smart meters are manufactured and programmed to the requirements of the OBIS code, so they have components with low resistance in handling both hardware and software. In case an electricity meter is fraud, it no longer sends to the network operator the actual energy consumption, but sends recordings that are much smaller than the real ones, so the non-invoiced energy registers as OTC of the energy distributor. In order to recover the respective electricity losses, the distribution operator applies a series of measures by which it identifies the consumption points with significant energy losses. The method applied by the distribution operators, in order to identify the defective or fraudulent measurement groups, is to perform annual energy balances on each area of the power distribution substations. After identifying the areas of consumption with a high percentage of losses, each individual consumer in the field has to be checked individually, operation that implies a high expense for the distribution operator and an embarrassing situation for the honest consumers.

In order to narrow the search area of the points where energy is lost, some distribution operators have installed some electronic devices called Feeder Remote Terminal Units (FRTU), which are able to detect the energy consumption of the downstream receivers and send the recorded data in real time to a server where an analysis is made for each receiver separately [38]. The installing of FRTUs narrows the problem groups search area but does not exactly identify the point or points where the power meter erroneously registers or electricity is stolen by other methods. For a most efficient exploitation of FRTUs, a series of algorithms can be implemented in order to restrict the fraudulent consumption points search area. In [39] is presented an algorithm of FRTUs functioning optimization which is designed on the probability of natural failure or by unauthorized intervention on intelligent power meters.

Most electricity distribution operators choose non-hardware solutions for detecting uncontrolled energy leaks, as they are more economically reliable, but they can only be implemented in areas where smart measurement systems are implemented. Of the recent non-hardware methods used in the detection of fraudulent consumption are those related to artificial intelligence; based on previous recorded consumption, a neural network [40] or a decision tree [41] can be created so that the difference between the consumption recorded by the power meter and the estimated one by the algorithm exceeds a certain limit, then at that point exists the possibility of an anomaly. One of the efficient methods for detecting consumers who steal electricity is to classify them using an Support Vector Machine (SVM), based on historical data related to electricity consumption and events recorded by the intelligent measurement system. In [42] is presented an algorithm based on SVM, which is trained with historical data of the monitored consumers, but also with a data set similar to a synthetic attack in order to control the energy consumed. In the process of classifying consumers, presented in [43], in order to identify consumption points with non-technical energy losses, the authors use a wide range of data from the electricity consumption history to data related to geographical positioning, as well as related to temperature and humidity. The results obtained using SVM algorithms are promising in the consumer areas where the remote management systems have been implemented for some time, thus there is a large database for each consumer partly related to both consumption and the events in the distribution network. The implementation of SM is topical and in process of development; many countries have few localities with intelligent systems of measurement and remote management implemented for at least five years, thus with designed algorithms based on data analysis to detect fraud in distribution systems considerable results cannot yet be achieved.

Algorithms based on data analytic, described in the above references, have an efficient application in monitoring of industrial consumers, where SM is fully implemented and diverse range of data is available. In the case of household consumers, it is necessary to apply some decision algorithms, which are based on a Boolean logic, or a fuzzy decision system, such as the one described in [44,45], where it is specified that NTL occurs not only at consumers with a tendency to decrease consumption, but also frequently in the case of consumers with a significant increase in consumption.

### 3.2. Smart Metering Communication Subsystem Implementations

Within the Electricity Distribution Operators there is a great diversity of technologies for the implementation of remote meter reading system as shown in Figure 2, starting from current loop communications systems, to systems based on GSM/GPRS communication or communications whose support is the power line, Power Line Communication (PLC). Due to the economic profitability [46] the PLC is currently one of the most adopted communication solutions. Besides the economic reasons mentioned, the PLC system has spread widely and due to its good acceptance among those who manage and implement the smart metering systems. In order to identify this degree of profitability, even studies have been carried out in this regard, an example being given by the paper [47] where the authors developed their own methodology which indicated that the best degree of adoption is represented by the PLC solution.

## 4. Smart Metering System Operation Optimization Solution

### 4.1. Mathematical Model Formulation of the Optimization Problem

The paper intends to develop an optimization solution for identifying the nodes in a system of electricity distribution, in which the consumed energy is not recorded correctly. In this sense, the aim is to develop and solve an optimization model, which is composed of: smart meters, gateways and the physical power support that is used as a communication medium. A series of factors such as: voltage drops in the nodes, data history, anomalies in the remote management systems, the existence of sporadic consumers, differences between the active energy and the reactive energy consumed, which can lead to the identification of the nodes with erroneous energy records were considered in developing the optimisation model. Thus the resulting optimization model proposed by authors is given by the cost function:

(1) $$\begin{array}{cc} F(\epsilon_{r},\alpha_{\Delta u},\beta_{W},\tau_{Wr}) & =M_{p}\left(\epsilon_{r}\right)+V_{p}\left(\Delta_{u}\right)+ERP_{p}\left(\beta_{W}\right)+ED_{WhVARh}\left(\tau_{Wr}\right)\\ \; & =\sum_{j=1}^{n}(\sum_{i=1}^{m}{\left(k_{i}\cdot c_{i}\cdot \epsilon_{rij}\cdot W_{i}\right)+\Delta E_{Tj}-W_{sj})}^{2})+\sum_{i=1}^{k}{\left(\alpha_{\Delta_{u}}\cdot \left(\Delta U_{i}+U_{i}\right)-U_{n}\right)}^{2}+\\ \; & +{\left(\beta_{Wi}\left(t\right)\cdot W_{i}\left(t\right)-W_{ierp}\left(t\right)\right)}^{2}+{\left(\tau_{Wri}\cdot W_{ir}\left(T\right)-W_{i}\left(T\right)\right)}^{2}) \end{array}$$

subject to:

(2) $$\begin{array}{c} \alpha_{\Delta ui},\beta_{Wi}\geq 1.\\ \Delta U_{i}\leq \frac{1}{100}\cdot U_{n}.\\ W_{i},W_{isap}\leq 0.\\ |\epsilon_{ri}|\leq \epsilon_{SMCi}\cdot W_{i}.\\ \epsilon_{ri}\neq 0. \end{array}$$

where:

*n*—number of transformation stations;

*m*—number of consumers from j transformation station area;

*k*—total number of consumers from the analyzed consumption area;

$M_{p}\left(\epsilon_{r}\right)$—the metering function precision expressed as $\epsilon_{r}$ optimization vector variable dependence;

$V_{p}\left(\Delta_{u}\right)$—the power failure, term expressed as $\Delta_{u}$ optimization vector variable dependence;

$ERP_{p}\left(\beta_{W}\right)$—the difference between measured electricity and registered in database, term expressed as $\beta_{W}$ optimization vector variable dependence;

$ED_{WhVARh}\left(\tau_{Wr}\right)$—the energy difference between reactive and active power.

$k_{i}$—coefficient determined based on previous experiences;

$c_{i}$—coefficient describing node i degree of connection (connected or disconnected);

$\epsilon_{ri}$—describes a measuring error of power meter between −0.5% and +0.5% in the case of household consumers;

$\epsilon_{SMCi}$—represents the value indicated by the precision class of the smart meter;

$W_{i}$—node i measured energy;

$\Delta E_{Tj}$—estimated technical energy loss;

$W_{s}$—electricity measured by the general power meter from the transformation station;

$\alpha_{\Delta u}$—coefficient determined by the voltage drop registration error on each node;

$\Delta U_{i}=\frac{\sum_{i=1}^{n}\left(R_{i}\cdot P_{i}+X_{i}\cdot Q_{i}\right)}{U_{n}}$—represents the calculation of the voltage drop in the electrical connection for each node;

$P_{i}$—the active power of consumer related to node *i*;

$R_{i}$—the electrical resistance of the electrical connection corresponding to the consumer *i*;

$X_{i}$—the electrical reactance corresponding to the connection of each node separately;

$Q_{i}$—the reactive energy recorded by each power meter;

$U_{n}$—nominal voltage;

$\beta_{Wi}$—error coefficient of data transfer between the telecommunication system and the data storage system; $W_{i}\left(t\right)$—the active energy recorded in node i at time t energia; $W_{isap}\left(t\right)$—the energy registered in the database of the billing information system reported at time *t*;

$\tau_{Wri}$—error coefficient, difference between the active energy and the reactive inductive energy in a analyzed time interval T.

$W_{ir}$—the reactive energy consumed, read at time *t*.

The optimization problem has been modeled starting from the facilities and the multitude of data offered by the remote reading systems implemented so far. The proposed objective is to track real-time energy consumption records from each node, comparing them to general measurement group records.

### 4.2. Blind Sparky Algorithm for Optimized Operation of an Electricity Utility Smart Metering System

Currently, the identification and correction of OTC is performed by manual analysis consisting in the differences between the energy delivered to the consumer and the one entered identification. This procedure based on the identification of inconsistencies in the energy balance indicates the existence of measurement errors that must be examined and identified individually and manually.

This procedure involves high costs and long time, as well as discomfort for the consumers. In order to eliminate these shortcomings, an algorithm is proposed which, based on the data provided by the smart metering system and a consumption model, will identify the nodes in which problems with the energy consumption or recording are registered.

The conceptual structure of the proposed solution is presented in the Algorithm 1.
**Algorithm 1:** Blind Sparky algorithm for maintaining smartmeters’ recordings consistency.
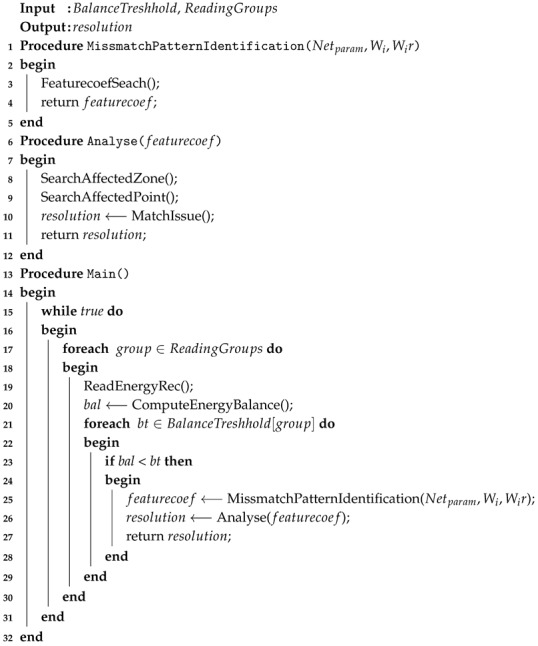


## 5. Experimental Case Study

The first step in detecting non-technical losses in a particular area is to foresee an energy balance for that area. If the respective balance is below a theoretical threshold calculated by the distribution operator taking into account the characteristics of the consumption area, then a series of technical measures should be taken in order to reduce the OTC.

An effective measure and without significant implementation costs is the verification of each measurement group in the respective area. Table 2 summarizes the energy balance data for a community in the Mures County—Romania for the year of operation 2019, before applying the optimization algorithm proposed in this paper.

**Figure 3:**
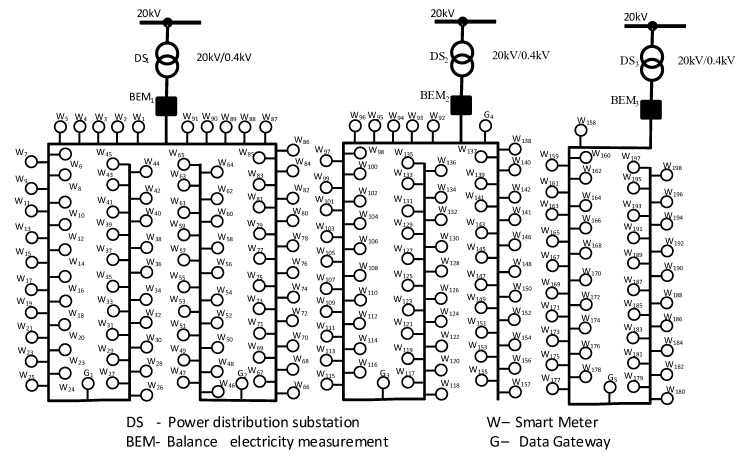
Smart metering system topology.

As it can be seen in Table 2, in the analyzed network with parameters given in Table 3 and topology shown in Figure 3, the energy losses are quite significant, even though the electricity network has been modernized and in the entire locality a PLC (Power Line Communication) system is implemented. The conclusion is that as much of the energy loss can found in activities such as unauthorized energy theft or in problems with some measurement groups.

Blind Sparky algorithm was applied to the entire measurement area, but especially to the power distribution substation areas $PTA_{1}$ and $PTA_{2}$, where the most significant energy losses were detected in order to identify fast and in the most accurate way erroneously registered measurements of electricity in certain nodes.

The successfully implementation of the described algorithm is conditioned by an adequate data communication assurance between the devices in the area $PTA_{1}$ and the two data concentrators located in the area of the respective substation. An important factor in the communication endangerment is the large distance between devices and gateway, as well as the harmonic pollution, so to increase the readability of the data, the algorithm described in the paper [48] has been implemented. Thus the information from the electricity meters located at a greater distance from the gateway is read by using neighboring devices (with the role of repeaters).

After the data acquisition related to active, inductive and capacitive energy consumption, as well as to the events and to the each node characteristic voltages, the data are processed by an application developed in the Python programming environment based on the Blind Sparky algorithm. The application provides for each node the values of the 4th coefficients described in the mathematical model: $\epsilon_{r},\alpha_{\Delta u},\beta_{W},\tau_{Wr}$.

If no anomaly is registered in a node, then the coefficients and the objective function will have the following values:

(3) $$\begin{array}{c} \epsilon_{r},\alpha_{\Delta u},\beta_{W}=1.\\ \tau_{Wr}\geq 1.\\ F(\epsilon_{r},\alpha_{\Delta u},\beta_{W},\tau_{Wr})\to 0. \end{array}$$

**Figure 4:**
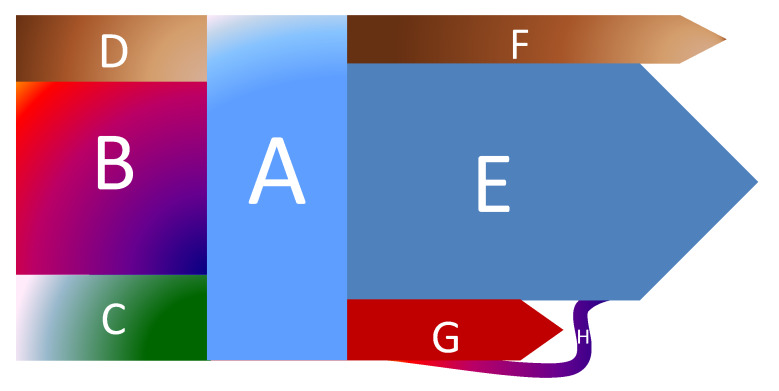
The energy outline at the power distribution grid level. A—Available Energy, F—Output exchange energy, E—Net energy delivered to consumers, D—Distribution grid interconnection, B—Electric power transmission interconnection, C—Local produced energy, G—OTC, H—Recovered component.

In Figure 4 is synthesized the concept of energy contour of an electricity distribution operator, containing the input energy (sections B, C and D, the energy distributed to the consumers (section A), the energy delivered to another distribution operator (section F) and the energy losses (OTC) (section G). Part of the OTC energy can be recovered by identifying the defective measurement groups, this quantity being invoiced according to the legal provisions (section H). Each distribution operator monitors daily the evolution of the energy contour, establishing the forecast of the energy exchanges for the next day and trying by using the methods described in this paper to reduce the technical losses, but especially the non-technical losses. An important element in balancing the energy contour is to increase the amount of recovered energy and to stop the losses of electricity from unauthorized interventions.

In the case of an energy meter corresponding to a node with a measurement error greater than the threshold imposed by the legal measurement rules, then the value of the coefficient $\epsilon_{r},$ will be greater than the unit and the value of the objective function will be greater than zero. There are many situations, especially in the disadvantaged areas, where certain consumers, after being disconnected from the electricity network, will connect themselves back illegally. In these situations, the consumed energy can’t be invoiced and the index from the telemetering system will be different from the index registered in the billing system ($\;{beta}_{W}>1$). The classic methods used to bypass the electricity meter, by false columns or shunts made under the terminal cap of the meter, will be detected by following the changing values of the coefficients $\;{alpha}_{\Delta u},\;{tau}_{Wr}.$

The algorithm described, based on the recorded data related to the consumption and on the technical data corresponding to the electrical network, calculates the value of the voltage drop related to each node, by using Equation (4):

(4) $$\Delta U_{i}=\frac{i\cdot (\rho_{MC}\cdot \frac{l_{MC}}{S_{MC}}\cdot \sum_{j=1}^{n}P_{j}+x_{MC}\cdot l_{MC}\cdot \sum_{j=1}^{n}Q_{j})}{U_{n}}+\frac{\left(\rho_{SC_{i}}\cdot \frac{l_{SC_{i}}}{S_{SC_{i}}}\cdot \sum_{j=1}^{n}P_{j}+x_{SC_{i}}\cdot l_{SC_{i}}\cdot \sum_{j=1}^{n}Q_{j}\right)}{U_{n}}$$

where: *i*—the number of connection point on given branch;

$\rho_{MC}$—electrical resistivity of the power line;

$l_{MC}$—length of the main electrical cable on a section;

$S_{MC}$—section of the main electrical cable;

$P_{j}$—active power;

$x_{MC}$—reactance of the main electrical cable;

$Q_{j}$—reactive power;

$U_{n}$—nominal voltage;

$rho_{SC_{i}}$—electrical resistivity of the branch;

$l_{SC_{i}}$ —length of the branch;

$S_{SC_{i}}$—section of the branch;

$x_{SC_{i}}$—reactance of the branch.

The obtained values are compared with the values recorded by the smart metering devices, so if a big difference is recorded between the two values, then in the respective node a suspicion of incorrect registration can be reported. In the case of false columns in most situations the inductive energy passes through the measurement group so the energy recorded on the 1st and 4th quadrants will be higher than the active energy recorded.

**Figure 5:**
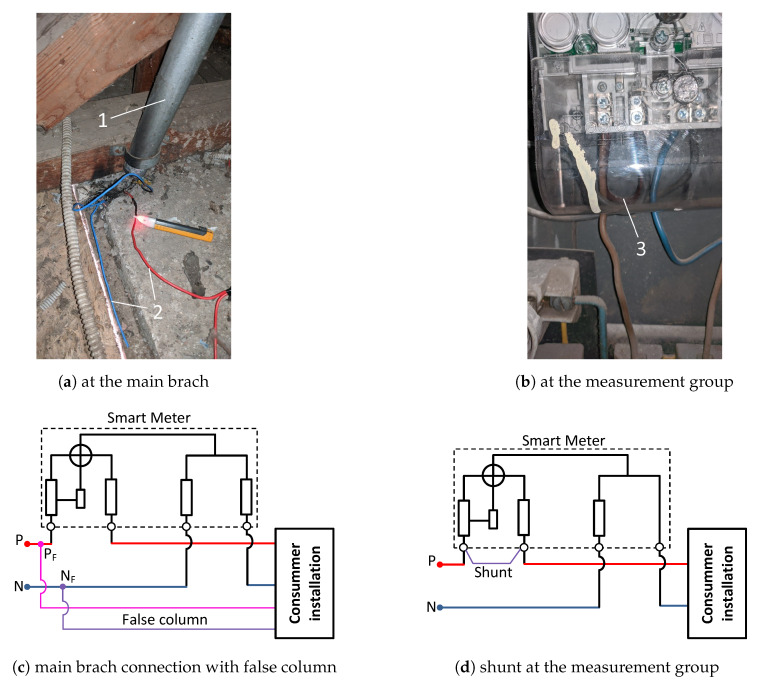
Unauthorised connection and their scheme (1—main branch, 2—false column, 3—shunt connection).

By applying the Blind Sparky algorithm on the analyzed measurement area, a series of irregularities were detected in some measurement groups, from meters with precision class malfunctioning, to well masked illegal installations mounted before the electricity meter, as presented in the Figure 5a,c which are dangerous for both the distribution operator and the consumer. Unauthorized installations do not comply with any norms from the point of view of fire prevention and extinction, as well as from the safety of the operating personnel. A more subtile method of stealing electricity is the one shown in the Figure 5b,d which consists of inserting a shunt between the input and output terminals of the electricity meter.

The Blind Sparky algorithm is able to detect this anomaly as well as the situation in which one of the consumers illegally reconnects to the electricity grid.

In the measurement area in which the algorithm presented in this paper was tested, four cases of possible fraud were detected as shown in Table 4, which were confirmed by an on-the-spot verification by the personnel from the distribution operator, in total being recovered an amount of 11,282 [kWh] according to the norms imposed by NERA (National Energy Regulatory Authority).

**Figure 6:**
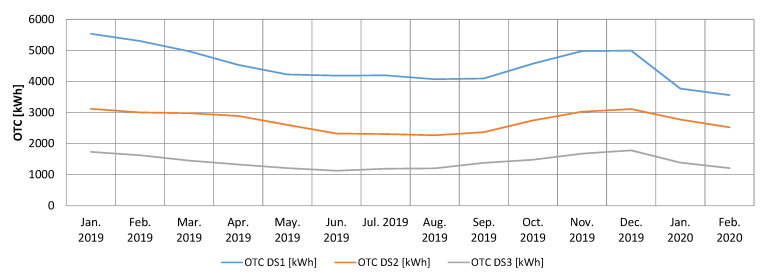
OTC evolution for January 2019–February 2020 per individual sector.

**Figure 7:**
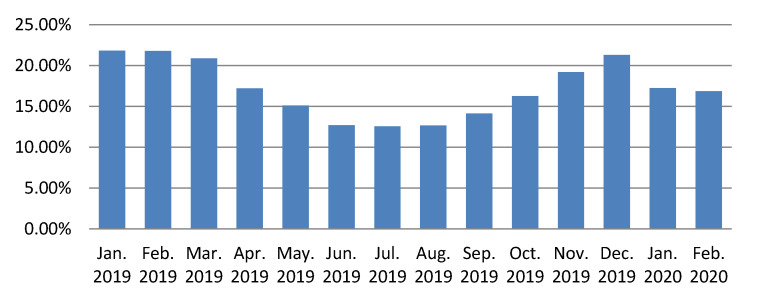
Own Technological Consumption (OTC) evolution in the case of the whole smart-meter based energy measurement analyzed sector.

In the Figure 6 is represented the evolution of the OTC, on three power distribution substations considered representative in the measurements analysis area. After the implementation of the described Blind Sparky algorithm, a significant decrease can be observed in the amount of OTC energy corresponding to the three power distribution substations. The implemented algorithm uses all the facilities offered by the electricity measurement sensors in each node, thus any anomaly related to the precise measurement of the electrical energy is instantly detected. The energy input and consumption of each node is reported every 15 min to the central system by the measurement sensors attached to each node, so the energy balances are updated continuously, any anomaly being detected in real time. The graph in the Figure 7 shows a decrease of about four percent of the OTC for the entire measurement analysis area, related to the total amount of distributed energy.

The Blind Sparky algorithm can be implemented on a much larger scale than the situation presented in this paper. It can be extended to all areas within the SDEE Mures where the intelligent measurement system is implemented. In the hypothesis that all the power meters within SR Mures would be able to provide real-time data for the implementation of the algorithm described in this paper, then there would be the possibility that the value of the OTC would decrease from approximately four percentages during the colder seasons to approximately two percent in the spring-summer period according to Table 5.

Energy forecast to be recovered throughout 2019 out of a total of approximately 156,000 MWh of losses reaches 1560 MWh.

**Figure 8:**
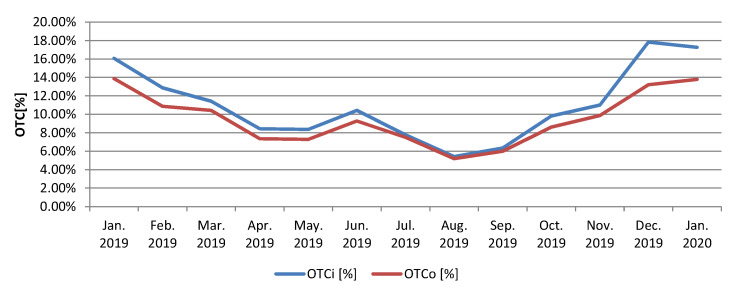
Measured and optimized OTC evolution for whole sector integrating analysed sector (OTCi—initial/measured values; OTCo—optimized values).

In Figure 8 is presented the comparative evolution of the recorded losses and of the losses resulted after optimization as a result of the sources of losses identification through the proposed algorithm.

## 6. Conclusions

Reducing the technical and non-technical losses of electricity is a priority for the power distribution operator motivated by the need to operate and provide the services under conditions of energy efficiency, economic competitiveness and compliance with national regulations and international standards.

Apparently less visible, the reduction of non-technical losses in the low voltage networks brings a financial benefit to the distribution operator, but also a significant improvement of the quality of the distributed electricity by adapting the consumption to the real needs and the projected capacity of the electricity supply system.

In this paper, a new algorithm for identifying locations with significant non-technical losses in a power distribution grid manifested in the form of incorrectly counted electricity is proposed. The proposed solution in this paper, called Blind Sparky, starts from an optimization algorithm that identifies the nodes with energy losses through the smart metering system. The proposed model includes multi-criteria aspects related to technical measurement errors and incidents, respectively non-technical ones, coming from altering the operating mode of the measuring systems in order to correctly bill the consumed electricity.

To validate and highlight the performance of the proposed algorithm, it was tested in a locality with 198 nodes within SR Mures. The results obtained in the test configuration illustrate that in a smart remote management system, by applying the proposed algorithm the non-technical losses can be significantly reduced, which leads to a significant improvement of the OTC especially during the winter period, when the fraudulent consumption of energy increases significantly. After the application of the Blind Sparky algorithm, some nodes where the electricity was not metered correctly in the presented configuration were identified, so after a spot check of the consumers indicated by the algorithm, a series of irregularities were detected and were remedied, being recovered, according to NERA norms, an amount of approximately 11 MWh, which reaches 10% of the total energy losses in the respective locality.

The algorithm presented and tested in the paper being validated for a single locality where smart remote management system is implemented, but the proposed solution can be extended as a functionality for the entire topology of low voltage networks within SR Mures. In this case the calculations show that there is the possibility of recovering non-invoiced consumptions around 1560 MWh which represents 1% of total losses related to the registrations for the year 2019.

Implementations of the smart metering systems brings many benefits from the consumer point of view such as more precise billing, flexible billing programe or awareness of the electricity consumption. However, significant benefits arise also for electricity distribution companies towards improving the quality of service and, if evaluated from the current work goal point of view, smart metering systems constitute base framework support for real-time monitoring solutions needed for high quality of the electricity delivery along identification of the non-technical losses sources.

## Figures and Tables

**Table 1:** Smart metering adoption advantages.

Electricity Distribution Operator	Electricity Supplier	Electricity Consumer
Accurate electricity consumption measurement	Recorded data real-time access	Hourly load curve visualization
Different events real-time notification	Billing errors reduction	Monthly billing
Accurate OTC forecast	Next day accurate energy demand forecast	Electricity consumption evaluation and improvement

**Table 2:** The 2019 energy balance for the considered investigated area.

Energy Balance Area	Number of Clients	Electricity Input [kWh]	Distributed Electricity [kWh]	OTC Electricity [kWh]	OTC Achieved Percentage [%]
$PTA_{1}$	91	302,873	247,205	55,668	18.38%
$PTA_{2}$	66	192,594	159,854	32,740	17.56%
$PTA_{3}$	41	119,901	102,732	17,169	14.32%
Total at substations level	198	615,368	509,791	105,577	17.15%

**Table 3:** Main parameters of the considered power distribution grid elements.

Power Distribution Element	$\rho \;[\frac{\Omega \cdot {\mathrm{mm}}^{2}}{m}]$	$x\;[\frac{\Omega }{\mathrm{km}}]$	l[m]	$S\;[{\mathrm{mm}}^{2}]$
Main section	0.015	0.33	40	3 × 70 Al + 1 × 50 Al
One-phase short branch	0.017	0.31	10–20	16 Al + 25 Al
One-phase long branch	0.018	0.31	20–50	16 Al + 25 Al
Three-phase branch	0.016	0.33	15–50	3 × 16 Al + 1 × 25 Al

**Table 4:** Results analysis.

Detected Affected Area	Affected Node	Detected Issue	Detection Coefficients Values	Amount of Recovered Electricity [kWh]	Electricity Saving Interval
$PTA_{1}$	11	False column (Figure 5a)	$\epsilon_{r},\;\beta_{W}=1,$ $\alpha_{\Delta u}=1.33,$ $\tau_{Wr}=1.23$	6575	13.08.2019–15.01.2020
$PTA_{1}$	23	Unauthorized re-connection	$\epsilon_{r},\;\alpha_{\Delta u},\tau_{Wr}=1,$ $\beta_{W}=3$	1725	20.10.2019–20.01.2020
$PTA_{2}$	121	Shunt under the terminals cap (Figure 5b)	$\epsilon_{r},\;\beta_{W}=1,$ $\alpha_{\Delta u}=1.47,$ $\tau_{Wr}=1.37$	2257	07.07.2019–20.01.2020
$PTA_{3}$	171	Unauthorized re-connection	$\epsilon_{r},\;\alpha_{\Delta u},\;\tau_{Wr}=1,$ $\beta_{W}=2$	725	22.11.2019–20.01.2020

**Table 5:** Experimental measured data and optimized results.

Analysed Period	The Amount of Electricity within a Contour [MWh]	The Amount of Distributed Electricity [MWh]	OTC Amount of Electricity [MWh]	OTC Percentage Achieved [%]	Forecasted OTC after Blind Sparky Algorithm Implementation [%]
January	155,786	134,218	21,567	16.07	13.87
February	140,603	124,566	16,036	12.87	10.87
March	148,577	133,338	15,239	11.43	10.43
April	136,056	125,470	10,586	8.44	7.36
May	137,113	126,537	10,576	8.36	7.29
June	135,258	95,263	19,930	10.42	9.28
July	148,631	113,540	8822	7.77	7.5
August	143,108	135,765	7343	5.41	5.2
September	135,116	127,062	8054	6.34	6
October	144,810	131,865	12,945	9.82	8.62
November	189,372	125,515	13,818	11.01	9.87
December	198,345	118,369	21,112	17.84	13.21
YEAR 2019	2,137,641	1,491,507	156,029	10.46	9.97

## Abbreviations

The following abbreviations are used in this manuscript:

AMI	Advanced Metering Infrastructure
FRTU	Feeder Remote Terminal Unit
IoE	Internet of Everything
IoT	Internet of Things
NERA	National Energy Regulatory Authority
NTL	Non-technical Losses
OBIS	Object Identification System
OTC	Own Technological Cosumption
PLC	Power Line Communication
SM	Smart Meter
SVM	Support Vector Machine
